# Rapid and simple detection of methicillin-resistance *staphylococcus aureus* by *orfX* loop-mediated isothermal amplification assay

**DOI:** 10.1186/1472-6750-14-8

**Published:** 2014-01-24

**Authors:** Jianyu Su, Xiaochen Liu, Hemiao Cui, Yanyan Li, Dingqiang Chen, Yanmei Li, Guangchao Yu

**Affiliations:** 1 College of Light Industry and Food Sciences, South China University of Technology, Guangzhou 510640, China; 2 State Key Laboratory of Food Science and Technology, Jiangnan University, Wuxi 214122, China; 3 Department of Laboratory Medicine, First Affiliated Hospital of Guangzhou Medical College, Guangzhou 510120, China; 4 Guangzhou Women and Children’s Medical Center, Guangzhou 510623, China; 5 Department of Microbial Pathogenesis, University of Maryland, Baltimore 21201, USA; 6 First Affiliated Hospital of Jinan University, Guangzhou 510620, China

**Keywords:** Loop-mediated isothermal amplification (LAMP), MRSA, *OrfX*

## Abstract

**Background:**

Methicillin-resistant *Staphylococcus aureus* (MRSA) has become one of the most prevalent pathogens responsible for nosocomial infections throughout the world. As clinical MRSA diagnosis is concerned, current diagnostic methodologies are restricted by significant drawbacks and novel methods are required for MRSA detection. This study aimed at developing a simple loop-mediated isothermal amplification (LAMP) assay targeting on *orfX* for the rapid detection of methicillin-resistance *Staphylococcus aureus* (MRSA).

**Results:**

The protocol was designed by targeting *orfX*, a highly conserved open reading frame in *S. aureus*. One hundred and sixteen reference strains, including 52 Gram-positive and 64 Gram-negative isolates, were included for evaluation and optimization of the *orfX*-LAMP assay. This assay had been further performed on 667 *Staphylococcus* (566 MRSA, 25 MSSA, 53 MRCNS and 23 MSCNS) strains and were comparatively validated by PCR assay using primers F3 and B3, with rapid template DNA processing, simple equipments (water bath) and direct result determination (both naked eye and under UV light) applied. The indispensability of each primer had been confirmed, and the optimal amplification was obtained under 65°C for 45 min. The 25 μl reactant was found to be the most cost-efficient volume, and the detection limit was determined to be 10 DNA copies and 10 CFU/reaction. High specificity was observed when *orfX*-LAMP assay was subjected to 116 reference strains. For application, 557 (98.4%, 557/566) and 519 (91.7%, 519/566) tested strains had been detected positive by LAMP and PCR assays. The detection rate, positive predictive value (PPV) and negative predictive value (NPV) of *orfX*-LAMP were 98.4%, 100% and 92.7% respectively.

**Conclusions:**

The established *orfX*-LAMP assay had been demonstrated to be a valid and rapid detection method on MRSA.

## Background

As a group of Gram-positive bacteria, staphylococci strains are responsible for various tissues infection and a wide range of diseases, including skin infections, pneumonia, endocarditis, osteomyelitis, gastroenteritis, scalded skin syndrome and toxic shock syndrome [1-4]. Within species of staphylococci, *Staphylococcus aureus* has been considered to be a major concern in both medicine and food safety [1-3,5,6]. For other clinically significant *Staphylococcus* strains, coagulase-negative staphylococci (CoNS) strains have been reported as the most frequently isolated pathogens in intravascular catheter related infections (CRI) (accounting for approximately 28% of nosocomial bloodstream infection), thus become a frequent cause of nosocomial infection and bacteremia, especially in patients with indwelling medical devices [7-9]. Since the first report in 1961, methicillin-resistant *Staphylococcus aureus* (MRSA) has become one of the most prevalent pathogens responsible for nosocomial infections throughout the world, which has raised a global challenge for clinicians, hospital epidemiologists and administrators [10-16]. The *mecA* gene, encoding the PBP2a protein and causing methicillin resistance in staphylococci, is located on staphylococcal cassette chromosome *mec* (SCC*mec*). SCC*mec* contains the *mec* gene complex (the *mecA* gene and its regulators) and the *ccr* gene complex (encoding site-specific recombinases responsible for the mobility of SCC*mec*) [17-23]. In recent years, MRSA strains have been considered to be a major example of the leading “Super Bugs”, with broad resistance to practically all β-lactam antibiotics and usually other multiple drugs due to the associated resistance genes carried by SCC*mec*[1-3,17-20]. As consequence, the increasing awareness for the risk and hazard of MRSA strains raised the demands of an early, cost-effective, timely, specific and sensitive detection assay [5,6,10,20].

As clinical MRSA diagnosis is concerned, *Staphylococcus* strains have commonly been identified via routine standard procedures including colony morphology, Gram staining, testing of catalase, hyaluronidase and coagulase, as well as the Vitek 2 automated system and the API-Staph commercial kit, which makes the detection of 16SrRNA somehow irrelevant. However, despite the advantages of this assay, the further development and broad application of multiplex-PCR has been restricted by many factors, such as cross-reaction of different sets of primers, self-inhibition due to formation of dimmers, reduced amplification efficiency caused by the simultaneous and parallel amplification, undetectable influence of different targets, as well as the requirement for standard full use of external and internal quality control (both positive and negative control for each targets) for each assay. As a novel analytical assay since the past decade, mass spectrometry has been applied for detection and diagnostics of various clinical microorganisms, with advantages on high-throughput process, sensitivity and specificity [24]. However, this methodology requires trained personnel, operating space, complicated sample preparation procedure, as well as expensive equipment.

**Figure 1:**
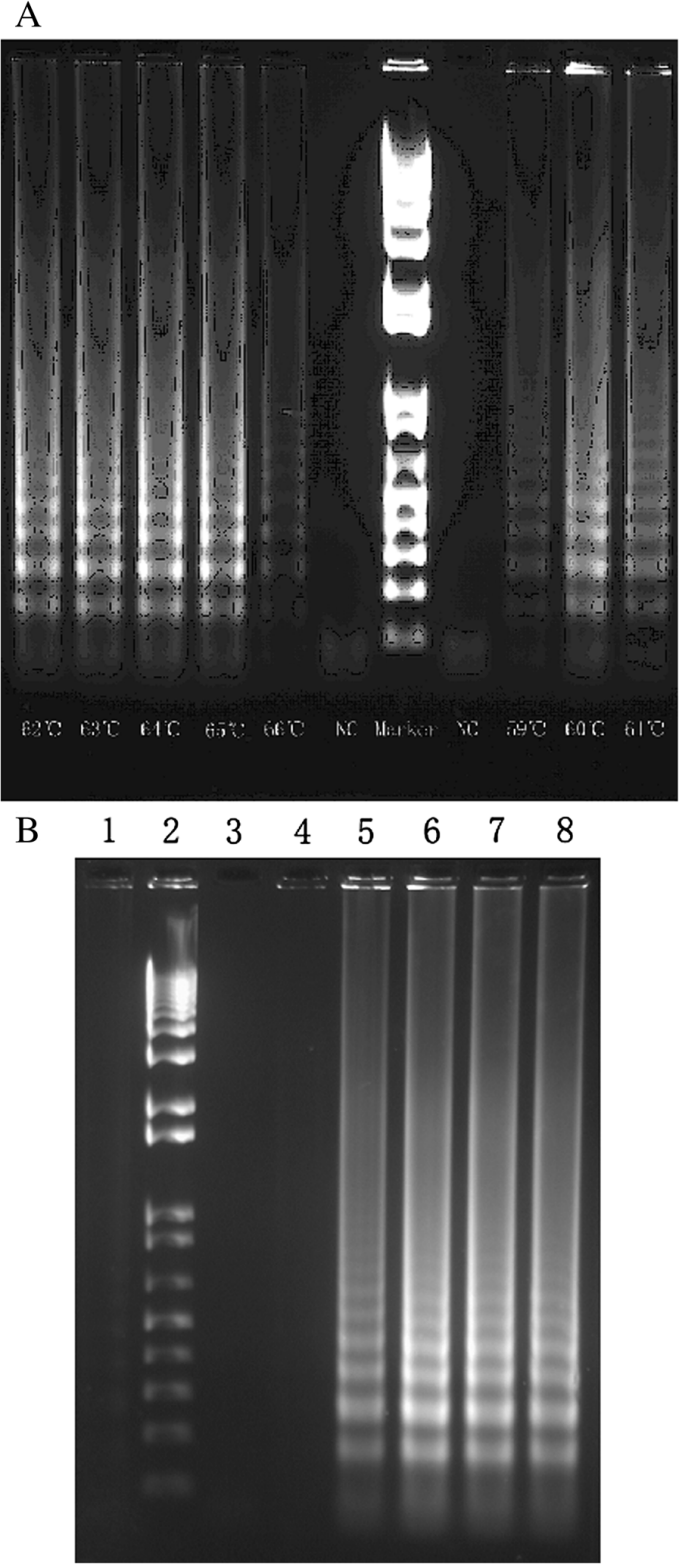
**Electrophoresis of *orfX*-LAMP. A**: Under different temperatures. **B**: Detection limit, lane 1-8: negative control (distilled water), DNA Marker, negative contro1 (template DNA sample of other bacteria), 1 copies, 10 copies, 10^2^ copies, 10^3^ copies and 10^4^ copies.

In the last decade, loop-mediated isothermal amplification (LAMP) had been reported as a novel nucleic acid amplification method [25-27] (Figure 1) and applied to the detection of various pathogenic organisms, including *Escherichia coli* O157 with the associated toxins *stx1* and *stx2*[28], *Yersinia pseudotuberculosis*[29], *Salmonella*[30-33], *Vibrio parahaemolyticus* with the associated toxins *tdh* and *trh*[34-39], *Psuedomonas aeruginosa*[40] and *Listeria monocytogenes*[41]. The LAMP methodology relies on an auto-cycling strand displacement DNA synthesis performed by the Bst DNA polymerase large fragment under the isothermal conditions between 60-65°C, and the amplicons are mixtures of many different sizes of stem-loop DNAs with several inverted repeats of the target sequence and cauliflower-like structures with multiple loops, which significantly simplify the reaction itself and the result determination. The LAMP assay includes 4 or 6 primers (targeting on 6 or 8 specific regions) to perform the amplification, and requires 45-60 min and 30-45 min without and with loop primers, respectively. In comparision with PCR assay, the positive points of LAMP method were simplicity, rapidity, specificity and sensitivity (10-1000 times higher than PCR).

In the current study, a simple *orfX*-LAMP assay had been developed, evaluated, optimized and further applied to detect a large scale of clinical MRSA strains, requiring approximately 60 mins for the whole process.

## Results

### Optimization of *orfX*-LAMP assay

**Figure 2:**
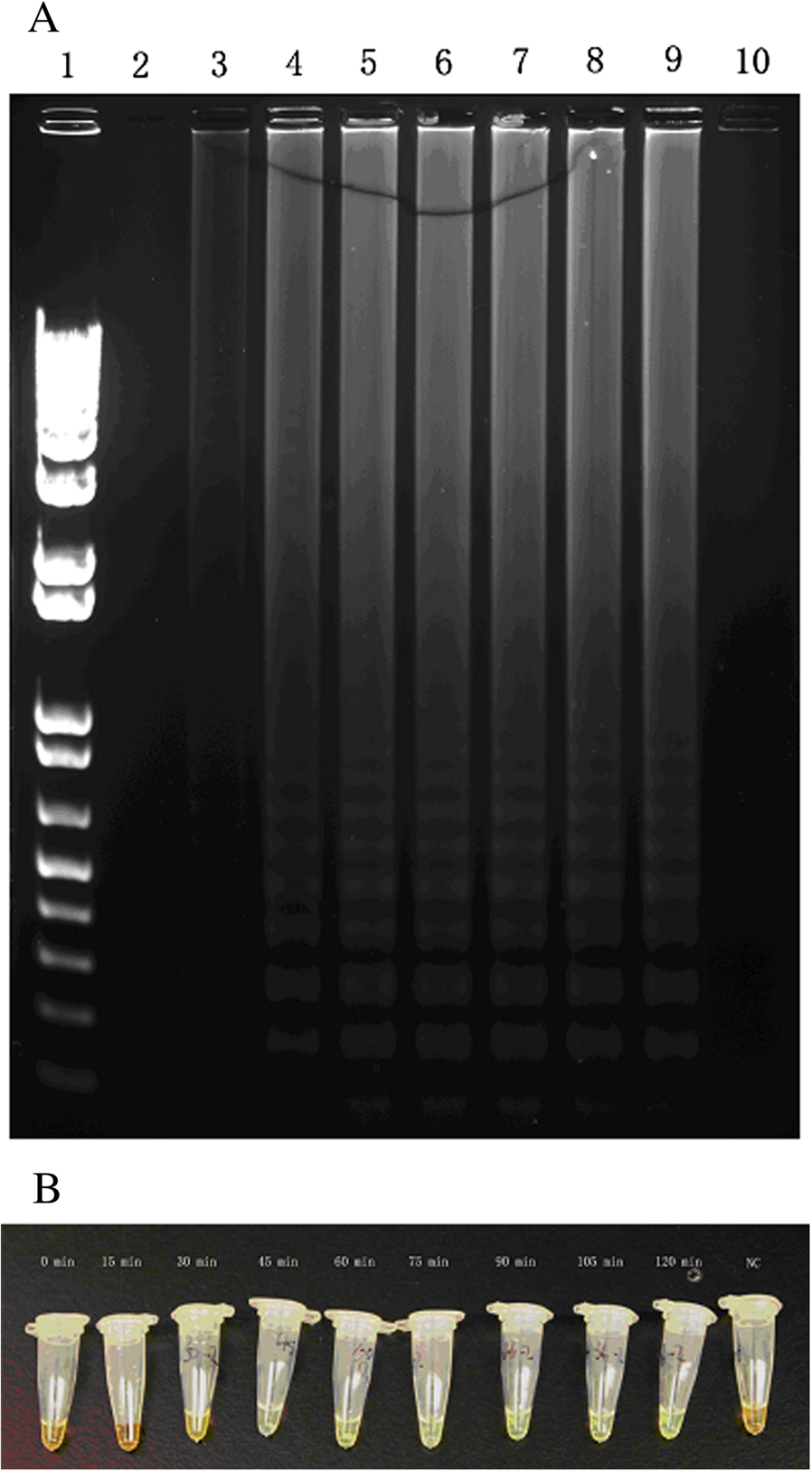
**Evaluation of *orfX*-LAMP assay under different time points. A**: Electrophoresis of *orfX*-LAMP reaction under different time points: 1-10: DNA Marker, 15, 30, 45, 60, 75, 90, 105 and 120 min, negative control. **B**: Sybr Green of *orfX*-LAMP reaction under different time points.

**Figure 3:**
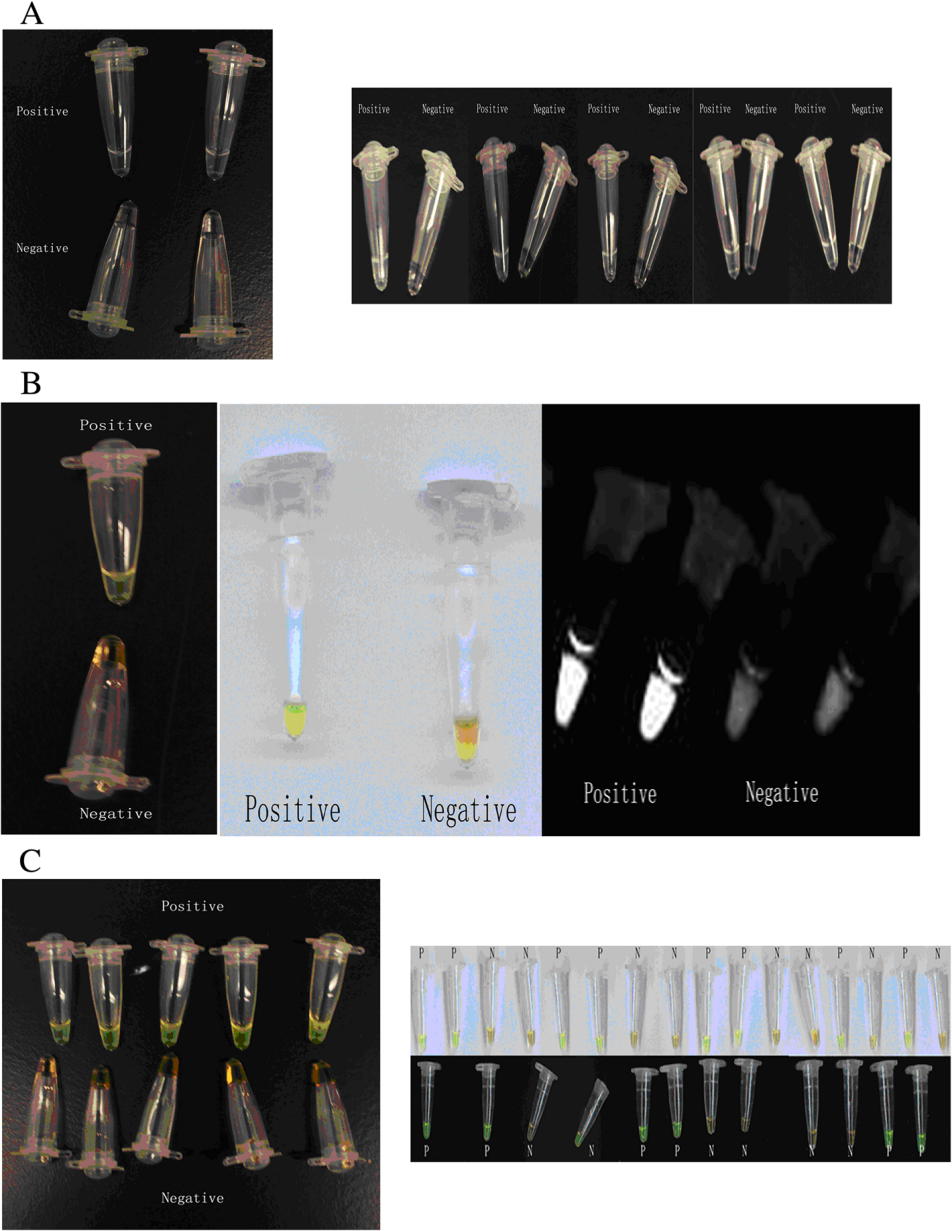
**Result determination of *orfX*-LAMP assays by color change. A**: Results determination of *orfX*-LAMP by naked eyes. **B**: Results determination of *orfX*-LAMP by Sybr Green with dark background, light backgrounds and under UV light. **C**: Results determination of *orfX*-LAMP in application by Sybr Green.

The *orfX*-LAMP amplification had generated a large number of ladder-like pattern bands on agarose gel due to the characteristic structure up to the loading wells, with a bottom band of 212-bp size amplicon obtained by sequencing. *OrfX*-LAMP assay had been performed under isothermal condition between 59°C and 66°C. Despite none of significant difference observed, the electrophoresis of LAMP products which were amplified under 64°C exhibited slightly larger amount of DNA amplicons when compared to other temperatures (Figure 1), which was consistent with studies previously. As reaction length was concerned, various time points had been studied under 65°C and with 10 ng template DNA, including 15 min, 30 min, 45 min, 60 min, 75 min, 90 min, 105 min and 120 min. For the LAMP assay with loop primers, amplification had been initially detected at 30 min, and reached the maximal at 45 min (Figure 2). Nevertheless, without loop primers, amplification products could not be observed until 90 min. All amplicons of *orfX*-LAMP assay had been determined by electrophoresis, with observation directly by naked eye, as well as under UV light with Sybr Green dyed (Figure 3).

### Sensitivity and specificity of *orfX*-LAMP assays

The detection limit of *orfX*-LAMP assay had been studied by both minimal CFU of bacterial and template DNA amount, which was found to be 10 copies of DNA/tube and 10 CFU/reaction (LAMP was positive for sample containing 1X10^4^ CFU/ml, with 1 μl was included in the reaction system) (Figure 1), in comparision with the detection limit of PCR which was 10^2^ copies of DNA/tube and 10^3^ CFU/reaction, indicating that the sensitive of LAMP was 10-100 times sensitive than PCR. As specificity was concerned, for primers, amplification could not be obtained in the absence of FIP, BIP, F3 or B3, due to the indispensable role each of the primers plays in auto-cycling strand placement. The LAMP was only performed in the existence of both inner primers and outer primers. In addition, for the *orfX*-LAMP assay, high specificity was acquired when this assay was subjected to 116 reference strains, without any false positive amplification for non-MRSA strains (Table 1).

**Table 1:** **Reference strains included in the evaluation of *orfX*-LAMP**

**Reference strains**	**No.**	***orfX***
Gram-positive microorganisms		
Methicillin-resistant *Staphylococcus aureus* 10442, COL, N315, 85/2082, CA05, JCSC 1978, JCSC 4469, MR108, M03-68, WIS	10	+
Methicillin-sensitive *Staphylococcus aureus* ATCC6358, ATCC12598, ATCC12600, ATCC13150, ATCC13565, ATCC14458, ATCC19095, ATCC23235, ATCC27664, ATCC25923, ATCC29213	11	-
Methicillin-resistant coagulase-negative staphylococci: *S. epidermidis* ATCC29887, ATCC700586	2	-
Methicillin-sensitive coagulase-negative staphylococci: *S. epidermidis* ATCC12228, 6508, 9142, 9142-M10; *S. haemolyticus* 1922; *S. hominis* ATCC27844; *S. capitis* ATCC27840; *S. saprophiticus* ATCC35552; *S. sciuri* ATCC29062; *S. schleiferi* ATCC43808; *S. intermedius* ATCC49052	11	-
*Listeria monocytogenes* ATCC15313ATCC19111, ATCC19114, ATCC19115, ATCC19116, ATCC19118, WF 03213, WF 06410, WF 08211	8	-
*Listeria invanovii* ATCC19119, WF 06319	2	-
*Listeria welshimeri* WF 05086	1	-
*Listeria seeligeri* WF 04426	1	-
*Bacillus cereus* ATCC11778, ATCC12480, ATCC13061, ATCC14579, ATCC25621, ATCC53522	6	-
Gram-negative organisms		
*Escherichia coli* O157: H7 WF01201 ATCC43889, NCTC12900	3	-
*Escherichia coli* O157: H7 WF07803, WF06544, WF05395	3	-
*Escherichia coli* O157: H7 WF04402, WF05311, WF06837	3	-
*Escherichia coli* O157: H7 WF04587, WF08385, WF06349	3	-
*Escherichia coli* O26: H11 WF054489	1	-
*Escherichia coli* O127: H6 WF073522	1	-
*Escherichia coli* O148: H28 WF063224	1	-
*Escherichia coli* C600 ATCC25922, ATCC43984, ATCC43985, ATCC8739, C600, DH5α	6	-
*Vibrio parahaemolyticus* O1: K25 WF 04213	1	-
*Vibrio parahaemolyticus* O1: K56 WF 02314	1	-
*Vibrio parahaemolyticus* O3: K6 WF 04232	1	-
*Vibrio parahaemolyticus* O3: K6 WF 01031, WF 04506, WF 06215	3	-
*Vibrio parahaemolyticus* O3: K6 WF 02321, WF 03218, WF 05612	3	-
*Vibrio parahaemolyticus* O3: K12 WF 02108	1	-
*Vibrio parahaemolyticus* O3: K72 WF 02471	1	-
*Vibrio parahaemolyticus* O4: K37 WF 01309, WF 04238	2	-
*Vibrio parahaemolyticus* O4: K55 WF 03256, WF 07521	2	-
*Vibrio parahaemolyticus* O4: K68 WF 07926, WF 03422, WF 02613	3	-
*Vibrio parahaemolyticus* O10: K28 WF 06438	1	-
*Vibrio parahaemolyticus* ATCC17802, ATCC27969	2	-
*Vibrio vulnificus* ATCC27562	1	-
*Vibrio mimicus* ATCC33653	1	-
*Psuedomonas aeruginosa* ATCC 27853	1	-
*Salmonella enterica* ATCC13076, ATCC14028, ATCC19585, ATCC29629	4	-
*Salmonella* typhimurium ATCC 14028, WF 04313	2	-
*Salmonella* choleraesuis ATCC 13312	1	-
*Salmonella* enteritidis WF 05148, WF 07086	2	-
*Salmonella* typhi WF 03201, WF 05026, WF 08138	3	-
*Salmonella* paratyphi WF 06426	1	-
*Salmonella* aberdeen WF 04542	1	-
*Salmonella* gallinarum WF 05938	1	-
*Klebsiella pneumoniae* ATCC 13883	1	-
*Enterobacter cloacae* ATCC 23355	1	-
*Yersinia enterocolitica* ATCC9610, ATCC27729	2	-
Total	116	

### Application of LAMP assays on clinical MRSA strains

After establishment and optimization, *orfX*-LAMP assay were applied to the detection of 667 clinical *Staphylococcus* strains, including 566 MRSA, 25 MSSA, 53 MRCNS and 23 MSCNS strains (Table 2), with comparative validation by standard PCR assay. For application, rapid DNA preparation process, simple heating equipments and results determination by observation directly by naked eye and under UV light had been applied. As shown by the results, for total of 566 MRSA strains, 557 (98.4%, 557/566) and 519 (91.7%, 519/566) had been detected positive by LAMP and PCR assays, respectively. However, for other 101 non MRSA isolates, none and 3 yielded positive amplification. The detection rate, positive predictive value (PPV) and negative predictive value (NPV) of *orfX*-LAMP were 98.4%, 100% and 92.7%, respectively. All reaction had been performed in triplicate to ensure reproducibility.

**Table 2:** **Application of the *orfX*-LAMP on clinical *Staphylococcus *** **strains**

**Samples**	**LAMP**	**PCR**
MRSA		
Bloodstream	100% (36/36)	94.4% (34/36)
Respiratory tract	98.6% (145147)	91.8% (135/147)
Skin and soft tissue	98.0% (238/243)	92.6% (225/243)
Urinary tract	98.6% (73/74)	91.9% (68/74)
Other	98.5% (65/66)	86.4% (57/66)
Subtotal	98.4% (557/566)	91.7% (519/566)
MSSA	0% (0/25)	0% (0/25)
MRCNS	0% (0/53)	0.0% (2/53)
MSCNS	0% (0/23)	0.0% (1/23)
Subtotal	0% (0/101)	0.0% (3/101)

Samples, Blood culture specimens included blood inoculated into aerobic and/or anaerobic blood culture medium. Tracheal secretion specimens included secretions from the trachea and bronchia and bronchoalveolar lavage fluid specimens. Wound specimens included surgical wound specimens but also specimens from ulcers, fistulae, abscesses, drainage fluids, catheters sites, percutaneous endoscopic gastrostomy insertion sites, and tracheostomas. Urine contained native urine from urinary catheters or bladder puncture and inoculated culture systems. Other specimens consisted of skin swabs specimens (from the nares, axilla, groin, or perianal area), as well as swab specimens of the throat, tonsils, eye, ear, and vagina; body liquids; puncture exudates.

## Discussion

As one of the globally widespread clinical pathogens, MRSA strains have attained a heightened concern for its rapid and accurate detection, which has become a common issue in the diagnostics of MRSA. Current MRSA diagnosis methodologies include routine standard procedures (including colony morphology, Gram staining, testing of catalase, hyaluronidase and coagulase), Vitek 2 automated system, API-Staph kit, immunological assays, mass spectrometry, PCR (regular PCR and quantitative PCR), etc. MRSA strains exhibit resistance to practically all β-lactam antibiotics and commonly other drugs due to the *mecA* and other resistance genes carried by SCC*mec*. However, for type I (1B) and IV (2B) SCC*mec* (with class B mec complex), the *mecI*-encoded repressor function may lead to the low-level methicillin resistance. In the case of type II (2A) and III (3A) SCCmec (with class A mec complex), the IS*431*-mediated deletion of *mecI* and further derepression of *mecA* transcription may cause the expression of methicillin resistance. As consequence, differing from biochemical methodologies, the accurate diagnostic identification of MRSA relies on the detection of highly specific and conserved targets within SCC*mec*. As *orfX* was considered to be highly specific target for both *S. aureus* and SCC*mec*, a distinctive MRSA detection methodology based on *orfX*-LAMP was developed in the present study. In addition, a number of attributes, including rapidity, simplicity in operation, specificity, sensitivity and expense, were also evaluated to verify the application of the *orfX*-LAMP assay for MRSA detection. Targeting on six distinct regions, inner and outer primers of the LAMP assay were highly specific in comparison with conventional PCR techniques. In this study, the specificity of the *orfX*-LAMP assay was verified by indispensibility of each primer, without any false positive test obtained from reference strains and 100% PPV for application. As sensitivity was concerned, in comparison with regular PCR (ranging from 10^3^-10^5^ CFU/reaction) [6] and previous LAMP detection methodoloy (ranging from 10^2^-10^3^ copies of DNA) [42], the detection limit of *orfX*-LAMP was found to be 10 copies DNA/tube and 10 CFU/reaction. For rapidity, approximate 60 min was required in the application of *orfX*-LAMP, including DNA process, isothermal reaction and result determination.

## Conclusions

The first study on detection of MRSA by LAMP assay was reported lately [6], however, 3 individual LAMP assays with targets including 16SrRNA, *femA* and *mecA* were laborious and time and expense demanding. Also, in another preliminary *Staphylococcus* LAMP identification assay, 120 min and 2 targets (*spaA* and *mecA*) were required for the procedure [42]. In this current study, a simple LAMP assay targeting on *orfX* for rapid MRSA detection had been developed, evaluated and optimized, which required approximately 60 min for each test and used the highly conserved target *orfX*. This *orfX*-LAMP had been further applied to the detection of 667 clinical *Staphylococcus* strains, and the sensitivity, specificity, PPV and NPV were found to be 98.4%, 100%, 100% and 92.7%, respectively. In comparison with conventional PCR, this established LAMP assay exhibited advantages on detection limit, sensitivity, simplicity and rapidity. In conclusion, this *orfX*-LAMP assay had been demonstrated to be a valid and rapid detection method on MRSA, which will undoubtedly aid in the broad application of bacteriological MRSA detection.

## Methods

### Bacterial strains

For development and evaluation of the *orfX*-LAMP assay, 116 reference strains were included, with various species of gram-negative and -positive isolates (Table 1). As application was concerned, the optimized LAMP and PCR assays was performed on a total of 667 *Staphylococcus* strains, including 566 MRSA, 25 MSSA, 53 MRCNS and 23 MSCNS strains (Table 2), which had been previously isolated from various clinical samples during 2001-2006 and preliminarily identified [6,9,15]. All strains were identified as *S. aureus* using standard procedures: colony morphology, Gram staining, testing of catalase, hyaluronidase and coagulase, the Vitek 2 automated system and the API-Staph commercial kit. Methicillin resistance was determined by susceptibility testing on oxacillin-screening agar, confirmed by latex agglutination for PBP2a and *mecA* detection by PCR [43].

### Primer design

The protocol of this LAMP assay for rapid MRSA detection was designed to target on the specific *orfX*, which located on the site of SCC*mec* and had been considered to be a highly conserved open reading frame in *S. aureus*. The sequences of orfX had been acquired on GenBank, including SCCmec type I (NCTC 10442, AB033763), SCCmec type II (N315, D86934), SCCmec type III (Mu50, gi:57634611), CA05, MR108, 86-3P, M03-6, 43000, SCCmec type V (WIS). A set of LAMP primers (Table 3) targeting 8 distinct regions on *orfX* was designed via PrimerExplorer (V4), including forward inner primer (FIP) with the complementary sequence of F1 (F1c), a T–T–T–T linker and F2, backward inner primer (BIP) with the complementary sequence of B1 (B1c), a T–T–T–T linker and B2, the outer primers F3 and B3 located outside of the F2 and B2 regions, loop primers LF and LB located between F2 and F1 or B1 and B2, respectively.

**Table 3:** The sequences and information of LAMP primers

**Label**	**5′pos**	**3′pos**	**len**	**Tm**	**5′dG**	**3′dG**	**GCrate**	**Sequence**
F3	204	222	19	56.03	-5.56	-4.06	0.42	ACCACAATCMACAGTCATT
B3	398	415	18	55.58	-7.53	-4.56	0.50	CCCGCATCATTTGATGTG
FIP			39					CAAAGTCGCTTTGCCCTTGG-GATGCTATCTTCCGAAGGA
BIP			41					GATCAAACGGCCTGCACAAG-GRAATGTCATTTTGCT RAATG
F2	243	261	19	55.33	-5.14	-4.71	0.47	GATGCTATCTTCCGAAGGA
F1c	291	310	20	61.82	-4.16	-5.45	0.55	CAAAGTCGCTTTGCCCTTGG
B2	377	397	21	55.47	-4.51	-4.07	0.38	GR(G or A)AATGTCATTTTGCTRAATG
B1c	326	345	20	61.55	-3.92	-4.66	0.55	GATCAAACGGCCTGCACAAG
LF	266	286	21	61.30	-6.24	-5.45	0.48	TGCGTTGGTTCAATTCTTGGG
LB	346	367	22	60.11	-5.26	-3.73	0.45	GACGTCTTACAACGCAGTAACT

### Establishment and optimization of *orfX*-Lamp assay

To evaluate and optimize the *orfX*-LAMP assay, 52 Gram-positive and 64 -negative isolates were employed as reference strains. Cultural conditions and DNA extraction of gram-negative and gram-positive strains were performed as described previously [41,44-47]. In brief, template DNA from *S. aureus* strains were prepared from overnight Tryptic Soy Broth (TSB) cultures at 37°C with shaking, and the collected culture was then diluted 10-fold in 10 mM Tris-HCl (pH 8.0) containing 1 mM EDTA. The suspension was boiled for 10 min and further kept on ice. After centrifugation at 12,000 *g* for 3 min, the resulting supernatant was used as templates for LAMP and PCR assays. Evaluation and optimization of this *orfX*-LAMP assay included the study of mixture volumn (4 volumns, with 12.5 μl, 25 μl and 50 μl), reaction temperature (8 temperatures, with 59°C, 60°C, 61°C, 62°C, 63°C, 64°C, 65°C and 66°C), reaction time (8 time points, with15 min, 30 min, 45 min, 60 min, 75 min, 90 min, 105 min and 120 min), specificity (including the primers and strains) and detection limit. LAMP assays was carried out in 3 different reaction mixture volumns, containing 1.6 μM (each) of the primers FIP and BIP, 0.2 μM (each) of the primers F3 and B3, 0.8 μM (each) of primers LF and LB, 1.6 mM of deoxynucleoside triphosphates, 6 mM MgSO_4_, 1 M betain (Sigma, St. Louis, MO, USA), 1 X thermopol buffer (New England Biolabs, Ipswich, MA, USA), and specified amounts of target genomic DNA. The reaction was initiated by heating at 95°C for 3 min, then chilled on ice for 30 sec, with 1 μl (8 U) of Bst DNA polymerase (New England Biolabs, Ipswich, MA, USA) further added. After incubation at various temperatures ranging from 59°C to 66°C for 15 min-120 min, the reaction was terminated by heating at 80°C for 2 min. As PCR assay was concerned, the amplification was carried out in 50 μl reactant, using the outer primers F3 and B3. The thermal profile was 94°C for 5 min, followed by 30 cycles of 94°C for 30 sec, 50°C for 30 sec, and 72°C for 30 sec and a final extension cycle at 72°C for 7 min. The amplified products (5 μl/well) were analyzed by gel electrophoresis in 2% agarose gels and stained with ethidium bromide for 10 min. Template DNA from MRSA 85/2082 was diluted for serial 10-fold and the detection limit of LAMP and PCR assays were ascertained by both minimal CFU of bacterial and plasmid DNA (with *orfX* recombinated in a T vector). For LAMP assays, the lowest bands from amplicons were sequenced by an ABI PRISM 310 genetic analyzer (PE Biosystems, Foster City, CA, USA).

### Application of LAMP assays on clinical MRSA strains

After optimization, the *orfX*-LAMP assay had been further performed on 667 *Staphylococcus* (566 MRSA, 25 MSSA, 53 MRCNS and 23 MSCNS) strains, and was comparatively validated by standard PCR assay. Template DNA was prepared through a rapid procedure as aforementioned, with the required time as less than 30 min. Heating and isothermal amplification were separately performed on simple equipments including water bath and heating block. Amplification products of LAMP assay were dyed with Sybr Green, positive or negative were determined through both visually observation of the color change by naked eye and a fluorescence assay under UV [6,28,33]. This experiment was performed in triplicate to ensure reproducibility.

## Abbreviations

LAMP: Loop-mediated isothermal amplification; MRSA: Methicillin-resistant *Staphylococcus aureus*; MSSA: Methicillin-sensitive *Staphylococcus aureus*; MRCNS: Methicillin-resistant coagulase-negative staphylococci; MSCNS: Methicillin-sensitive coagulase-negative staphylococci.

## Competing interests

The authors declare that they have no competing interests.

## Authors’ contributions

JS carried out the LAMP detection assays and drafted the manuscript. XL carried out the DNA preparation and participated in the bacterial processing. HC performed the statistical analysis. YL carried out the sample collection and bacterial processing. DC participated in the design of the study and revised the manuscript. YL conceived of the study and participated in its design and coordination. GY participate in the design of the study and manuscript revision. All authors read and approved the final manuscript.
